# Acute Bacterial Sacroiliitis With Associated Iliacus Abscess in a Healthy Young Woman: A Case Report

**DOI:** 10.1155/crdi/5942523

**Published:** 2025-11-03

**Authors:** Zahra Sheidae Mehne, Kiana Ketabi, Hoorieh Soleimani, Sadegh Ebrahimi, Elham Honarjou

**Affiliations:** ^1^Department of Infectious Diseases and Tropical Medicine, Mashhad University of Medical Sciences, Mashhad, Iran; ^2^Department of Radiology, Mashhad University of Medical Sciences, Mashhad, Iran; ^3^Department of Emergency Medicine, Mashhad University of Medical Sciences, Mashhad, Iran

**Keywords:** acute bacterial sacroiliitis, case report, immunocompetent adult, infectious arthritis, MRI, *Staphylococcus aureus*

## Abstract

**Background:**

Acute bacterial sacroiliitis is a rare yet potentially debilitating infection of the sacroiliac (SI) joint. While often associated with immunocompromised states, intravenous drug use, or trauma, this condition can also manifest in, otherwise, healthy individuals. Its nonspecific clinical presentation frequently mimics common musculoskeletal disorders, such as mechanical back pain or inflammatory arthropathies, leading to underdiagnosis and treatment delays. *Staphylococcus aureus* is the most prevalent pathogen involved, and without timely recognition and intervention, the infection can result in irreversible joint destruction. Therefore, early diagnosis is critical to prevent long-term disability and ensure favorable patient outcomes.

**Objective:**

This case report presents a rare instance of acute bacterial sacroiliitis in a previously healthy, immunocompetent adult female with no predisposing conditions. The report emphasizes the diagnostic challenges, the role of imaging, and the necessity for early intervention.

**Case Presentation:**

A 40-year-old woman with no significant medical history presented with a 21-day history of progressively worsening pain in the left SI joint, radiating to the left lower limb, accompanied by fever, anorexia, and chills. Initial treatment with analgesics and nonsteroidal anti-inflammatory drugs proved ineffective, and laboratory tests revealed significantly elevated inflammatory markers. Magnetic resonance imaging (MRI) demonstrated joint effusion, a multiloculated abscess within the left iliacus muscle, and adjacent myositis. Ultrasound-guided arthrocentesis confirmed *Staphylococcus aureus* as the causative organism. The patient was successfully treated with intravenous antibiotics, followed by oral therapy and surgical drainage.

**Conclusion:**

This case highlights the importance of maintaining a high index of suspicion for bacterial sacroiliitis in immunocompetent adults presenting with persistent pelvic pain. Prompt diagnosis through advanced imaging and targeted therapy is essential to prevent complications and ensure favorable clinical outcomes.

## 1. Introduction

Acute bacterial sacroiliitis is a rare but potentially debilitating infection of the sacroiliac (SI) joint, often underdiagnosed due to its nonspecific clinical presentation and low incidence [1, 2]. The condition results from microbial invasion, most commonly by *Staphylococcus aureus*, and can mimic other musculoskeletal disorders such as mechanical back pain, sciatica, or inflammatory arthropathies. This overlap can lead to diagnostic delays and, consequently, severe joint destruction [3, 4]. Although septic sacroiliitis is more commonly associated with predisposing factors such as immunosuppression, joint disease, or recent invasive procedures, it may also occur in, otherwise, healthy individuals [5, 6].

Even in the absence of classic risk factors, certain antecedent events—such as cesarean section—may indirectly alter the biomechanical environment or transiently affect host defenses, thereby predisposing patients to infection. Early and accurate diagnosis is crucial to prevent irreversible joint destruction and chronic disability. Magnetic resonance imaging (MRI) has emerged as the preferred imaging modality for early detection, given its high sensitivity in identifying joint effusion, bone marrow edema, and associated soft tissue abscesses that may not be visible on plain radiographs or computed tomography (CT) scans [7, 8]. Although blood cultures often fail to identify the causative pathogen, direct synovial fluid aspiration remains the gold standard for microbiological diagnosis and for guiding targeted antimicrobial therapy [9].

In this report, we present a rare case of acute bacterial sacroiliitis in a previously healthy, immunocompetent 40‐year‐old female who underwent cesarean section six months prior.

## 2. Case Presentation

### 2.1. Patient History and Presentation

A 40-year-old woman with no significant past medical history presented to the emergency department with a 21-day history of progressively worsening pain in the left hip and pelvic region. The pain radiated down her left leg and was accompanied by fever, severe chills, and anorexia. Initially, she managed her symptoms with analgesics and nonsteroidal anti-inflammatory drugs; however, her condition deteriorated to the point where weight-bearing became difficult.

### 2.2. Physical Examination

On evaluation, the patient was alert and oriented. Vital signs revealed a temperature of 38.1°C, pulse rate of 100 beats per minute, blood pressure of 110/70 mmHg, respiratory rate of 16 breaths per minute, and oxygen saturation of 98% on room air. Focused examination of the pelvic region demonstrated significant tenderness and swelling over the left SI joint. Both the Straight-Leg-Raise and FABER tests were positive, and there was marked restriction in the range of motion of the hip and SI joint. The remainder of the physical examination, including cardiac, pulmonary, and abdominal assessments, was unremarkable. The patient reported having undergone a cesarean section 6 months earlier and worked as a housekeeper; she denied any history of chronic illness or immunosuppressive therapy.

### 2.3. Laboratory Assessment

Laboratory investigations demonstrated a pronounced inflammatory response. Serial inflammatory markers were markedly elevated, with an erythrocyte sedimentation rate (ESR) peaking at 111 mm/hr and C-reactive protein (CRP) levels reaching 220.9 mg/dL prior to admission. Although these values fluctuated during hospitalization, they showed a gradual decline by the time of discharge, indicating a resolving inflammatory process. The white blood cell (WBC) count was also elevated, with neutrophil predominance observed throughout the course of illness (Table 1). Notably, Brucellosis serology, blood cultures, and synovial fluid cultures remained negative.

### 2.4. Radiological Assessment

**Figure 1:**
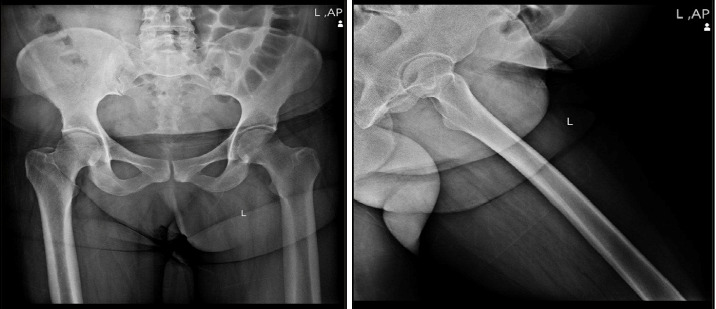
An X-ray examination of the patient, no erosions or bone destruction.

**Figure 2:**
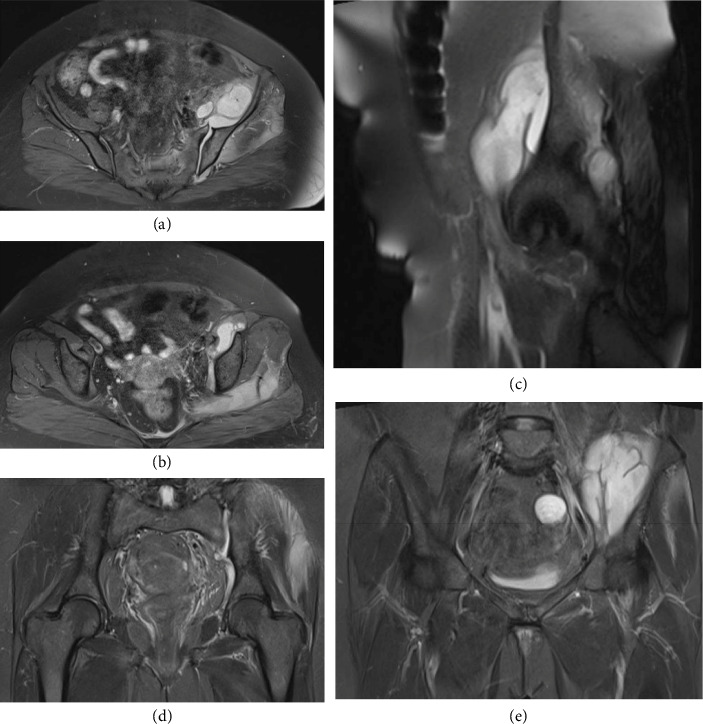
MRI study; (a-b) (axial STIR): sacroiliac joint effusion, iliacus abscess collection, and myositis involving the gluteus minimus and gluteus medius muscles. (c) Sagittal STIR: abscess within the iliacus muscle. (d) Coronal STIR: sacroiliac joint effusion and a normal hip joint. (e) Coronal STIR: iliacus abscess formation and an incidental simple cyst in the left ovary.

Given the clinical presentation and laboratory findings, imaging studies were performed. Ultrasound revealed a mild effusion in the left SI joint without evidence of abscess formation or tumoral lesions. Plain radiographs were unremarkable, showing no erosions or bone destruction (Figure 1). However, MRI provided critical diagnostic insight: it confirmed the presence of an effusion in the left SI joint and identified a multiloculated abscess within the left iliacus muscle, measuring 78 × 65 × 35 mm, which was contiguous with the SI joint effusion. Hyperintense signal changes consistent with myositis were observed in the left pelvic muscles, including the gluteus minimus and medius, obturator internus, and piriformis. Importantly, no erosions or bone destruction were detected in the SI joint, and no effusion was present in the hip joint; however, mild bone marrow edema was noted in the adjacent iliac bone (Figures 2(a), 2(b), 2(c), 2(d), and 2(e)).

### 2.5. Diagnostic Arthrocentesis

To establish a definitive diagnosis, an ultrasound-guided arthrocentesis of the left SI joint was performed under local anesthesia. Ten cubic centimeters of purulent fluid were aspirated. Analysis of the fluid revealed a turbid appearance, with 156,250 WBCs/mm^3^ (85% neutrophils) and 7000 RBCs/mm^3^ (Table 2). Gram staining identified Gram-positive cocci, and subsequent culture confirmed *Staphylococcus aureus*. The organism was sensitive to several antibiotics, including cephalexin, ciprofloxacin, clindamycin, doxycycline, erythromycin, gentamicin, and oxacillin, but resistant to sulfamethoxazole–trimethoprim (SXT).

### 2.6. Management and Outcome

Empiric intravenous vancomycin (1 g every 12 h) was initiated for 14 days to ensure coverage against potential MRSA until culture results were available. Following confirmation of antibiotic sensitivity and completion of a 2-week course of intravenous therapy, the patient demonstrated marked clinical improvement. She was then transitioned to oral cephalexin (500 mg every 6 h) to complete a six-week antibiotic regimen. In addition, she underwent surgical drainage via arthrotomy, during which thorough lavage of the joint and drainage of the multiloculated iliacus abscess were performed.

Within 48 h postoperatively, the patient experienced significant pain relief, normalization of body temperature, and improved joint mobility. Serial laboratory evaluations demonstrated a steady decline in inflammatory markers. At the 6-week follow-up, clinical examination confirmed complete resolution of the infection. At 3 months posttreatment, she remained asymptomatic with no evidence of recurrence.

## 3. Discussion

Acute bacterial sacroiliitis in immunocompetent individuals is a rare and diagnostically challenging condition [1].

This condition represents approximately 1%-2% of all septic arthritis cases, with an annual Incidence estimated at 1-2 per 100,000 population. Although uncommon, delayed recognition and treatment can lead to substantial morbidity. Bacteremia accompanies 50%–70% of the cases, while abscess formation—typically involving the iliacus muscle—complicates 20%–30% of patients with septic sacroiliitis [10].

*Staphylococcus aureus* dominates the microbiological landscape, accounting for 60%–80% of infections [11–13]. Other notable pathogens include Salmonella species (particularly in sickle cell disease), *Pseudomonas aeruginosa* (predominantly in intravenous drug users), *Streptococcal species*, *Brucella*, and *Streptococcus pyogenes* [14, 15]. Recent reports have broadened our understanding of atypical presentations: Kiskani and colleagues described an unusual case of sacroiliitis with endocarditis following afebrile diarrhea, demonstrating that fever may be absent [16]. Kumar's team documented *Salmonella typhi* causing iliopsoas abscess with concurrent sacroiliitis in an immunocompetent young man, underscoring the varied microbial etiology and the need to consider enteric pathogens when clinically appropriate [17].

In this case, a 40-year-old woman with no significant underlying medical history presented with progressive left SI joint pain, high fever, and systemic signs, which raised strong clinical suspicion for a septic process. The delayed onset of symptoms—occurring 6 months after an uncomplicated cesarean section—is unusual, as most reported cases typically manifest within a shorter interval following surgery or other precipitating events [2].

Advanced imaging, particularly MRI, played a pivotal role in delineating the extent of infection. Unlike plain radiographs and ultrasound, MRI not only confirmed the presence of a joint effusion but also revealed a multiloculated abscess within the left iliacus muscle and associated myositis. These findings are consistent with previous studies emphasizing the indispensable role of MRI in the early detection and accurate assessment of septic sacroiliitis [3, 4]. Similar to the case reported by Tamvakopoulos et al. [18], the significantly elevated inflammatory markers in our patient necessitated advanced imaging for definitive diagnosis. The unremarkable X-ray findings further highlight the limitations of conventional radiography in detecting early bony or soft tissue changes.

Furthermore, our case reinforces that the absence of a clearly identifiable source of infection—illustrated by negative blood cultures—does not exclude the possibility of septic sacroiliitis. Direct synovial fluid aspiration remains the gold standard for microbiological diagnosis, as demonstrated by the isolation of *Staphylococcus aureus* from our patient's joint aspirate [4, 19].

In our case, the causative organism was identified as methicillin-sensitive *Staphylococcus aureus* (MSSA), based on antibiotic susceptibility testing showing sensitivity to oxacillin. The initial empirical choice of vancomycin was appropriate given the increasing prevalence of community-acquired MRSA and the severity of presentation. However, once MSSA was confirmed, the optimal treatment would have been de-escalation to a beta-lactam antibiotic such as cefazolin or cloxacillin, as these agents demonstrate superior outcomes compared with vancomycin for MSSA infections [20, 21]. The decision to continue vancomycin in our patient was based on clinical improvement and concerns about medication changes during the critical phase of treatment, though we acknowledge that targeted therapy with a beta-lactam would have been preferable [22].

Comparative analyses with previous case reports further underscore the clinical relevance of our findings. Bindal and Krabak [6] described a middle-aged patient with similarly nonspecific symptoms and delayed diagnosis, while Nutcharoen et al. [19] reported a case of sacroiliitis following cesarean section under spinal anesthesia. More recently, Monteiro et al. [23] presented a case complicated by abscess formation that required both prolonged antibiotic therapy and surgical intervention.

Antibiotic treatment for a staphylococcal iliac abscess should be administered for 4 days after adequate source control is achieved (e.g., percutaneous or surgical drainage), according to the most recent recommendations from the Surgical Infection Society [24, 25].

This duration applies to both low- and high-risk patients, including those with sepsis or comorbidities such as diabetes and obesity, and is supported by randomized controlled trial data showing no difference in outcomes between short and longer courses after source control. If source control is not achieved, antimicrobial therapy should be continued for 5–7 days, with clinical parameters (resolution of fever, leukocytosis, and restoration of gastrointestinal function) guiding discontinuation. Persistent signs of infection beyond this period warrant reassessment for additional source control intervention [25]. For staphylococcal abscesses specifically, empirical therapy should cover MRSA if risk factors are present, with agents such as vancomycin or linezolid, and then tailored based on culture results [26, 27]. There is no evidence supporting longer durations unless ongoing systemic infection or inadequate source control is present. In summary, 4 days of antibiotic therapy after source control is the current consensus for staphylococcal iliac abscess, with extension to 5–7 days if source control is not achieved or clinical improvement is delayed. Due to joint involvement requiring surgical drainage, the final treatment period in this case was set at 6 weeks, including 2 weeks of intravenous antibiotics.

It is important to note that, despite the absence of traditional risk factors such as immunosuppression or chronic systemic disease, our patient developed a severe infection. This suggests that factors unrelated to the classic postpartum state—possibly including prior surgical history—may compromise the SI joint environment, rendering it susceptible to bacterial invasion. The human aspect of this case lies in recognizing that even in the absence of overt risk factors, persistent and severe musculoskeletal pain should prompt thorough evaluation for possible septic arthritis. Notably, Imagama et al. [28] presented a similar case of pyogenic sacroiliitis caused by MRSA, highlighting the emerging role of community-acquired MRSA and the importance of including anti-MRSA agents in empirical therapy when clinical suspicion is high.

A limitation of this report is the absence of follow-up imaging to objectively document complete resolution of the iliacus abscess after treatment.

## 4. Conclusion

This case clearly illustrates that acute bacterial sacroiliitis, though rare, can occur in immunocompetent individuals without conventional predisposing factors. The persistent and severe nature of pelvic pain—especially when accompanied by systemic signs such as fever and elevated inflammatory markers—should prompt clinicians to consider septic sacroiliitis in the differential diagnosis. Early diagnosis, facilitated by advanced imaging modalities such as MRI, along with timely intervention using targeted antibiotic therapy (with appropriate MRSA coverage when indicated) and surgical drainage, is crucial to preventing irreversible joint damage and ensuring favorable clinical outcomes. Ultimately, heightened clinical vigilance and a multidisciplinary approach are paramount in managing this potentially debilitating condition.

## Figures and Tables

**Table 1:** Trends of inflammatory markers and hematologic parameters before, during, and after hospital admission.

Parameter	7-days preadmission	3-days preadmission	Admission	7-days postadmission	Discharge
ESR (mm/hr)	71	107	111	110	105
CRP (mg/dL)	220.9	126.6	41.2	81.5	23
WBC (× 10^3^/μL)	19.48	26.20	17.2	20.2	7.33
Neutrophils (%)	80.5	79.2	81.4	90.6	69.1

Abbreviations: CRP, C-Reactive protein; ESR, erythrocyte sedimentation rate; WBC, white blood cells.

**Table 2:** Synovial fluid analysis results.

Test	Result
*Macroscopic analysis*	
Appearance	Turbid
Viscosity	Abnormal

*Microscopic analysis*	
WBC count	156,250 mm^3^
RBC count	7000 mm^3^
Polymorphonuclear cells	85%
Mononuclear cells	15%
Bacteria	Seen
Color	Milky

## Data Availability

The dataset supporting this article's conclusions is confidential and cannot be made publicly available. However, data may be available upon reasonable request and with permission of the original data providers, subject to compliance with applicable confidentiality agreements and data protection laws, via contact through the corresponding author.
